# A Real-Time Inspection System for Industrial Helical Gears

**DOI:** 10.3390/s23208541

**Published:** 2023-10-18

**Authors:** Thomas Idzik, Matthew Veres, Cole Tarry, Medhat Moussa

**Affiliations:** School of Engineering, University of Guelph, Guelph, ON N1G 1W2, Canada; tidzik@outlook.com (T.I.); mveres@uoguelph.ca (M.V.); ctarry@uoguelph.ca (C.T.)

**Keywords:** automotive gear inspection, deep learning, quality control

## Abstract

Manufacturing is an imperfect process that requires frequent checks and verifications to ensure products are being produced properly. In many cases, such as visual inspection, these checks can be automated to a certain degree. Incorporating advanced inspection techniques (i.e., via deep learning) into real-world inspection pipelines requires different mechanical, machine vision, and process-level considerations. In this work, we present an approach that builds upon prior work at an automotive gear facility located in Guelph, Ontario, which is looking to expand its defect detection capabilities. We outline a set of inspection-cell changes, which has led to full-gear surface scanning and inspection at a rate of every 7.5 s, and which is currently able to detect three common types of surface-level defects.

## 1. Introduction

Inspection and quality control are integral to the manufacturing pipeline. Manufactured objects, parts, and components can be diverse and have various shapes. There are also different criteria for what counts as a defect (which may lead to either rejecting the part as scrap or possibly being reworked) and for which parts of the manufactured product need to be inspected. While human inspection is typically more flexible and can quickly adapt to different conditions, the process is repetitive, and scaling human labor is expensive.

In prior work [1], a deep learning algorithm for inspecting industrial gears was proposed. The algorithm used data from a local automotive gear manufacturer located in Guelph, Ontario, Canada, and obtained promising results. However, in order to integrate this work into the production environment, there are several system-level challenges that must first be resolved in order to to achieve real-time inspection performance consistent with the existing production cycle time. Two of the largest obstacles are represented by the inspection cycle time, as well as the detection of multiple kinds of defects.

## 2. Background and Literature Review

### 2.1. Visual Inspection of Gears for Quality Control

Recent work has shown how visual inspection methodologies, including point-cloud generation and CAD matching [2,3], can be used for verifying gears are produced according to specification. Point cloud generation, alignment, and CAD matching can be a computationally expensive operation to perform. Our current investigation focuses on detecting and identifying surface-level defects on helical gears, which can be observed through visual RGB means alone and is currently performed by human operators on the factory line. We seek a vision-based system that can mimic the current human visual inspection workflow and learn and operate directly from RGB data. In this work, we target the use of deep-convolutional networks, which also have the added benefit of being able to use dedicated graphics processing units for acceleration.

### 2.2. Deep Learning for Defect Detection

In general manufacturing applications, the use of deep learning techniques for visual inspection is gradually becoming much more commonplace. It has been used, for example, in problems such as: steel surface detection (6 defects) [4], vehicle body paint inspection [5] (6 defects), power-train transmission inspection [6] (2 defects), bearing inspection [7] (4 defects), wheel hub inspection [8] (4 defects), and others [9] (1 defect). These works typically focus on defect detection from an algorithmic perspective. Extending detection to operate within real or existing industrial pipelines and environments requires many intricate considerations, including both the cycle time and overall system implementation. For an overview of recent methods within manufacturing, we refer the reader to [10].

Many of these considerations (including the number and types of defects to target) need to be tailored to the specific process itself. Focusing specifically on defect detection for gears, this problem has been framed as a multi-class detection problem for different defects on gear end-faces. For example, Yu et al. [11] have explored broken teeth, missing teeth, and surface scratch defects, while Su et al. [12] have explored scratches, dents, and bumps. In contrast to the full, 3D surface inspection of helical gears in our current work, however (which requires imaging both teeth and end-faces), detecting defects on the gear end-face can be conducted through only a single image per gear. Some works have also tackled defect detection of the surface of gear teeth, including pitting, as explored by [13]. As can be seen from these works, even for such a targeted detection methodology as defect detection on gears, there are a number of variations in the process, scope, and approach that necessitate targeted inspection algorithm development.

### 2.3. Prior Work

**Figure 1:**
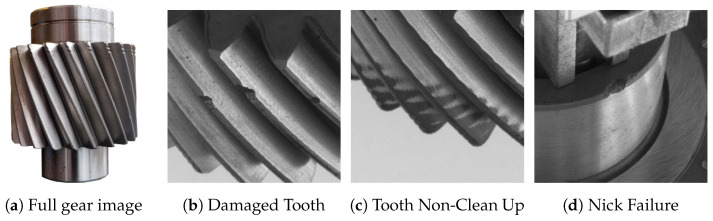
Sample automotive gear, along with the three defects explored in this work (**b**–**d**). Defects can occur anywhere on the gears’ surface. Note the grooves on the upper tooth portion of (**a**) are intentional and not a defect—they are used to quickly distinguish the type of gear.

This paper extends the defect detection algorithm presented in Allam et al. [1] as well as the data acquisition vision station developed by Hall [14]. Allam et al. proposed a method for detecting a defect known as damaged teeth, which can be seen in Figure 1b. The algorithm is based on a Faster-RCNN model [15]. When used for automated inspection of every tooth on every gear, it can reduce the inspection task of all non-defective gears (i.e., the bulk of manufactured parts) by 66% [1]. However, this algorithm only considered a single type of defect and was not optimized for speed. In this paper, we incorporate additional commonly occurring defects into the inspection procedure and optimize the inspection process speed such that it can be integrated within the existing gear manufacturing process.

### 2.4. Defect Descriptions

The data used in this paper were collected from a collaborating gear manufacturing plant. Among the possible defects, the plant identified damaged tooth (tooth), tooth non-clean up (ncu-teeth), and nick failure (nick) to be among three of the most commonly occurring types and are targeted in this work. These defects are characterized as follows:

- Damaged Tooth: A negative material defect in which significant damage has occurred to the faces of one or more tooth edges. This defect occurs on the inner or outer tooth faces or along the face of the tooth edge.
- Tooth Non-Clean Up: A positive material defect in which portions of the inner and outer tooth faces are incompletely ground clean from the initial casting surface. This defect appears as a series of non-polished regions towards the top or bottom edges of the tooth face.
- Nick Failure: A negative material defect in which the material has been dented, broken off, or damaged on the upper or lower journal. Nick failures occur on the edge between the top face of the upper journal and the upper journal face or the edge between the bottom face of the lower journal and the lower journal face.

Sample images of each defect can be seen in Figure 1. For a comprehensive list of possible defects at this facility, we refer the reader to [14].

### 2.5. Contributions

This article has two contributions. First, we present an integrated system for inspecting industrial gears, which includes mechatronic, machine vision, and machine learning components. The system is able to perform inspection (combined scanning and detection) at a rate of just 7.5 s per gear and is able to detect three different kinds of defects occurring on the gears’ surface. The system satisfies both operational and performance constraints and provides sufficient performance for wider adoption into existing quality control procedures.

Second, in a production environment, learning-based models need to be continually monitored and updated for a number of different reasons. Changes in the environment may affect feature representations, the system may need to be expanded to detect additional defects, or there may be a shift in the data distribution leading to a drop in performance. In this work, we explore how the effects of the physical imaging environment affects performance and the impact of labeled data size on defect inspection accuracy.

## 3. System Design

### 3.1. Automotive Gear Inspection Constraints

The objects inspected in this work are helical gears used in automotive systems. A sample image can be seen in Figure 1a. The factory produces four configurations of these gears, which primarily differ in the number of teeth they have, having either 22 or 26 teeth. Currently, human inspectors are being used to manually inspect each manufactured gear for signs of defects arising from the manufacturing process. In order to incorporate the system within the current quality control pipeline, the collaborating facility requires a system that is able to detect multiple types of defects, on any surface of the gear, at a minimum rate of 1 gear per 10 s (we refer to this time as our real-time constraint, as completing the inspection within this time period will have no impact on the existing process). The factory also requires components within the cell to be robust to movement, as the location of the cell may change from time to time. Other requested features include the operators’ ability to review past scans and inspection results.

### 3.2. System Overview

**Figure 2:**
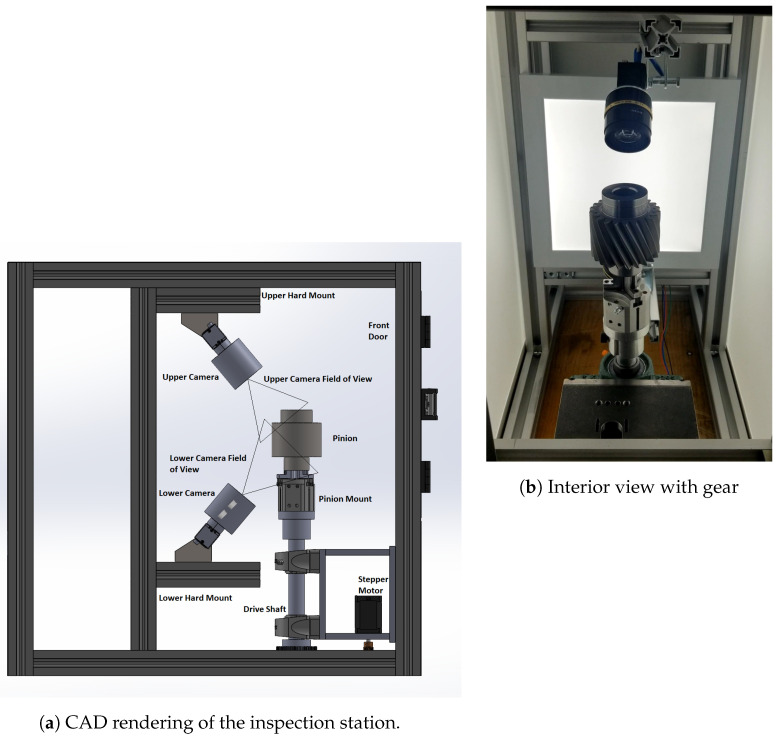
CAD rendering and physical cell overview. The upper and lower hard camera mounts referenced in the CAD drawing prevent camera movement during transportation.

The inspection cell design is presented in Figure 2a. The cell (Figure 2b) is a box-like structure that separates the imaging environment from the rest of the workplace and contains all required mechanical and machine vision hardware. A host computer runs a software stack composed of three main programs, each controlling different parts of the inspection process. One program runs the front-end HMI, another controls the collection and inspection of images, and the final one manages access to the scan database. The HMI is built as a web application using Javascript and Vue.

Within the inspection cell, two cameras are used to take images of the gear from opposing directions, and a pinion mount is used to rotate the gear continuously. The pinion mount is driven by a 17HS24-2104S NEMA 17 standard stepper motor [16], while the two cameras are Teledyne FLIR BFS-U3-27S5M-C cameras [17] with Tamron M23F08 lenses [18]. The camera types and specification represent a major change from the earlier station design reported in Grant [14] and used by Allam et al. [1], which prioritizes shorter exposure times with lower absolute sensitivity thresholds and a larger light capture surface area per pixel. Two 4400-lumen panels are attached to the left and right walls of the inspection cell, and a 1600-lumen panel is attached to the back of the cell behind the cameras and illuminates the gear. The cameras are mounted at an angle to reduce shadow effects on the gear and to allow for full imaging of the gears’ surface.

### 3.3. Inspection Cycle Optimizations

Using multiple cameras and continuous gear rotation during inspection is constrained by both image processing speed as well as by motion blur. Motion blur can be avoided by having a fast camera shutter speed; however, this reduces light and impacts image quality. To achieve real time image processing, the processing pipeline needs to be carefully fine-tuned. To facilitate this pipeline, we sourced a processor with 20 CPU threads and assigned these threads to different worker thread pools, handling individual parts of the inspection process. These thread pools are used for tasks such as: saving the images to storage, copying the image data to the GPU, scanning for defects, and transferring the detection results back to the main thread. A Controllino Mini is used separately for handling the gear rotation and inspection cell lights and is queried at the start of the inspection procedure. During development, we experimented with several different rotation speeds and found a minimum cycle time of 5 s to yield relatively high-quality images with minimal blurring. For more information on the system, please see [19].

## 4. Multi-Defect Gear Teeth Inspection

### 4.1. Multi-Class Defect Detection

The detection model proposed in this paper is an extension of the single-defect, Faster-RCNN model explored in [1], where three defects instead of only one are targeted during inspection. These defects are damaged tooth, tooth non-cleanup, and nick failure defects and are described in Section 2.4. We use a model to maintain consistency with prior work, as well as the overall ease of use and extensability of the implementation itself [20].

There are several ways that defect inspection could be extended from one to multiple types of defects. One way could be to maintain a single model per defect and to allow them to specialize in detecting that single defect only. If new defect types are encountered in the future, a new model would then be trained solely to detect that detect type alone—without requiring a complete re-training of previously trained models. A drawback, however, is that all models still require re-calibrating predictions and retraining periodically—including when additional data is available or when changes in the data are observed and detection performance decreases. Once the number of defects scales beyond a few, this task becomes resource intensive. In addition, as inspection would also require inference across all models for every image, there could also be an impact on the cycle time and possibly lead to a violation of the cycle time constraints.

Instead, in this work, we take the view that a single, multi-defect detection model could be more advantageous. Particularly, we focus on the notion that visual features and computation between classes can be shared and that the inference for different defects can be optimized as a single forward pass of the model. In this scenario, when adding additional defect classes to the model in the model in the future, there is likely to be a negligible effect on the inference time—translating into a constant inspection time across future detection algorithm iterations.

### 4.2. Data Collection

**Figure 3:**
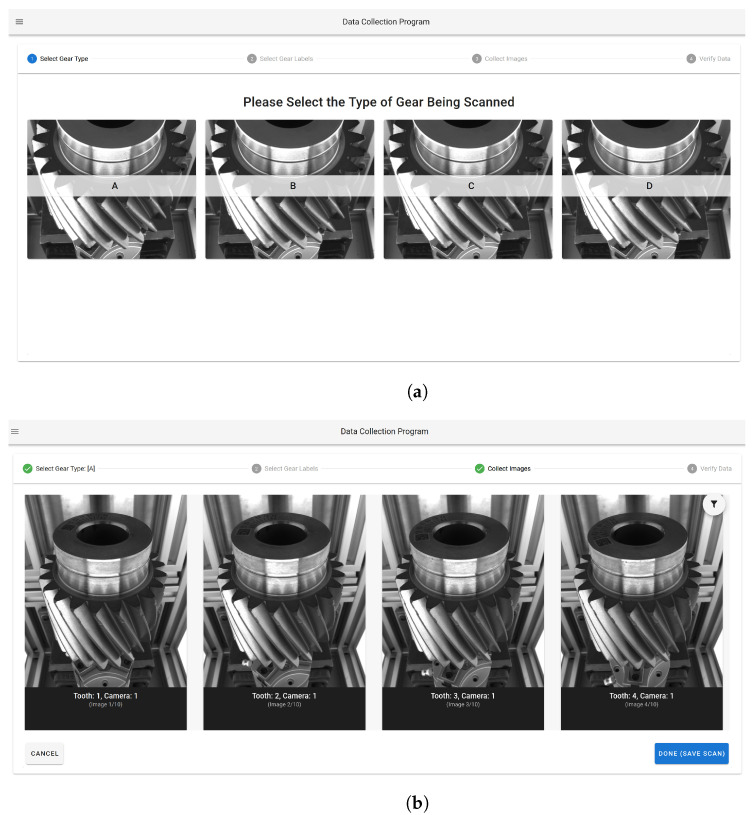
Different views from the HMI. The top bar in each view shows the current phase of the scanning operation. (**a**) Welcome Screen: The operator first selects the type of gear (A, B, C, or D), as well as relevant defect labels. The gear type is used to determine how many teeth (and thus images) need to be collected; type C is 26 teeth, while the others are 22. (**b**) Scanning and Inspection: Images and results are returned to the user. Information on each card includes the: Gear Tooth #, Camera #, Image # (out of 44 or 52 images), and any predicted defects.

The inspection system described in Section 3 was used to collect data in the form of gear scans, which were subsequently labeled for training the defect detection model. Whenever inspection personnel at the factory observed a defect on a gear, the type of defect was recorded, and the gear was set aside in a pile to be scanned later at the inspection station. Scanning begins with an operator loading gear into the inspection station. The operator then selects the type of gear being scanned on the HMI and adds any gear meta-data, which includes information on, e.g., any kinds of defects that human inspectors may have identified. This label is then considered as ground truth during data labelling. Sample views shown to the operator (via the HMI) can be seen in Figure 3.

During scanning, the gear is rotated continuously, and two cameras simultaneously collect an image of every tooth from both the top and bottom perspective. After scanning, images of scans with defects were labeled in an offline environment using Coco Annotator [21]. Table 1 shows an overall count of the number of gear scans recorded by our system to date. In addition to the defects targeted in this work, the “Other” category of Table 1 represents a set of 22 other defects that have been scanned by the inspection cell but that do not yet have a fair amount of samples available for learning and evaluation. As data has been collected continuously while improvements to the system were being carried out, image resolution between scans may vary slightly. In our dataset, our smallest image has a resolution of 1464 × 1936 pixels, while our largest image has a resolution of 2448 × 2048 pixels.

### 4.3. Updated Domain Knowledge Constraint

The prediction model operates on each image individually. However, since a defect can appear in multiple images as the gear rotates, Allam et al. [1] showed that using an additional constraint (in the form of confirming the detection of the same defect over a sequence of images) could improve performance. This constraint is given by a value ϕ, which specifies how many sequential images the defect must be detected in. In that work, the authors also applied the constraint against different prediction confidence thresholds from the model, which we denote here as *p*. We adopt the same domain-specific heuristic in this work and extend it to the multi-defect setting. The heuristic can be seen in Algorithm 1 and is applied to each camera independently. As we need to account for the cylindrical nature of the gear scan (i.e., any tooth can be the starting location and defects can wrap-around), in practice, we calculate this heuristic after all images have been inspected. Once this metric has been calculated, we can obtain a prediction for the entire gear.
**Algorithm 1:** Domain knowledge constraint**Data**: Inspected gear scan $S_{c}$ from camera *C***Given:** Sequential image threshold ϕ (1≤ϕ≤teeth)       Prediction confidence threshold *p* (0<p≤1)       List of known defects *D***Result:** Boolean: whether the gear contains a defect 
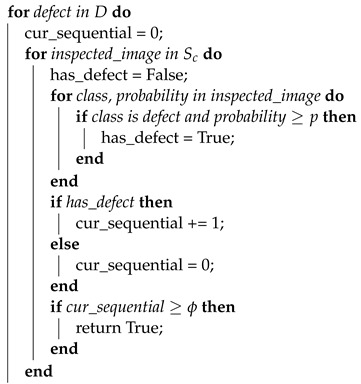
**end**return False;

### 4.4. Overall Inspection Procedure

**Figure 4:**
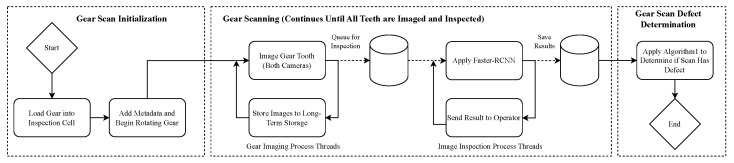
Overall inspection process diagram. During the Gear Scanning procedure, images are captured and added to a queue, where the Faster-RCNN model pulls from the queue and inspects the image for defects. These operations occur in parallel by using different thread pools.

Combining all components together, our overall inspection procedure can be seen in Figure 4. The user first loads the gear to be inspected and adds any metadata. Once the scan begins, a stepper motor rotates the gear at a fixed speed, and images of each tooth are taken by a top and bottom-facing camera. These images are then added to a queue for defect inspection. While the gear is rotating and images are being collected, the detection algorithm takes the first element in the queue and performs inspection. Results of this are saved to a database and also shown to the scan operator. Once all teeth have been imaged and inspected, Algorithm 1 is applied to the scan results to determine if a defect is present on the gear or not.

### 4.5. Training and Testing Datasets

The training dataset consists of images collected at different times, including damaged-tooth defects [1], nick defects [22], and a new set of labeled images having NCU defects. The distribution of labeled images and instances can be seen in Table 2. Note the comparatively high number of NCU instances (brackets) to the number of images.

The testing dataset reflects the real-world task of full-gear inspection and is composed of gear scans with either 44 or 52 images. Our testing set is split into two data sources: gear scans collected from an earlier inspection station design [14] and gear scans from the new design discussed in Section 3. For the original configuration, we use 15 gear scans of Tooth Non-Cleanup, 30 gear scans of Damaged Tooth, 30 gear scans of Nick Failure, and 99 gear scans of No Defect (note that one defect-free scan from [1] was removed due to a corrupted image in the scan). We also use a new set of 95 defect-free gear scans (as inspected by factory personnel) in order to evaluate generalization performance across imaging environments.

## 5. Experiments

Gear inspection in an industrial setting represents a dynamic environment. Changes in process parameters or machine wear and tear can impact the data entering inspection. Thus, it is important to evaluate not only the model accuracy in identifying gears with or without a defect, but to also understand how new or different data entering the training process affects the detection ability.

### 5.1. Experimental Setup

Our defect detection model is a pre-trained Faster-RCNN architecture, which is trained jointly on the three different types of defects. We build our model using Detectron2 [20] and use a ResNet-50 backbone. We refer to this pre-trained model as the “base model", which is re-trained from this state for all experiments. In all experiments, we fine-tune this model or 10,000 iterations using a batch size of 18 images on an NVIDIA GeForce RTX 3090. Images are resized to have a minimal side length of 800 pixels and maximum side length of 1333 pixels during pre-processing.

We measure our models’ performance on entire gear-scans using False Positive (FP) and False Negative (FN) metrics, taking the factory-inspected label as the ground-truth value. Note that FP indicates classifying a defect-free gear as having a defect, while FN indicates missing a defective gear.

### 5.2. Experiment 1: Multi-Defect Detection Model

**Figure 5:**
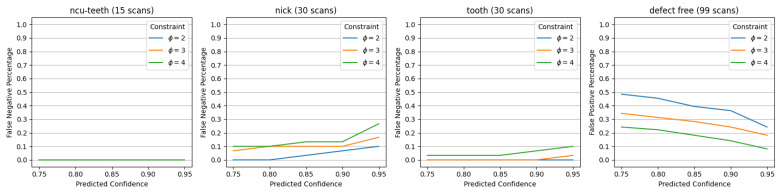
Model performance for defective and non-defective gears using FN and FP metrics and various inspection parameters.

In the first experiment, we seek to understand how well our model functions in its primary objective of detecting various defects on production gears. Figure 5 shows the False Negative results for each type of defect plotted against the predicted confidence threshold *p*. We also plot the performance under different sequential image constraints ϕ defined in Algorithm 1.

From the first three plots, it can be seen that the model can achieve a 100% accuracy in detecting NCU or Tooth type defects at a confidence threshold of p=0.9 and ϕ=3 sequential images to confirm the detection. We note that FN is used here since it is critical that the system does not miss any defects on any inspected gears. However, this high sensitivity can also lead to creating many false positive detections. Each false positive will lead to manual inspection of the gear, thus defeating the purpose of this inspection cell.

The last plot of Figure 5 shows the FP detections on defect-free gears given various detection thresholds. At a threshold of p=0.9 and using ϕ=3 consecutive images for confirming defects, the model has a 24.2% FP rate. This means that roughly 24% of all non-defective production gears will still be manually inspected. In terms of the sequential image constraint ϕ, it can be seen that increasing the constraint both (a) increases the number of false negatives predicted by the model and (b) decreases the number of false positives. Balancing this trade-off is key to organizing and optimizing downstream manual gear inspection tasks.

Finally, Figure 5 also shows that performance is not the same across all defect types. As shown in Figure 1, defects can have different visual features: NCU-teeth represents a rather large pattern that can typically be found across many teeth of a gear. The tooth defect can also typically be identified easily on the gear’s surface due to the contrast between the defect and surface of the gear. However, the nick defect remains a challenge. Both the size and amount of material that is missing can vary, and the upper and lower parts of the gear may face challenges with illumination.

### 5.3. Experiment 2: Data Requirements for New Defects

The three defects explored in this work are only a subset of all possible defects that can be observed at the gear manufacturing plant [14]. We expect that any deployed models will need to be expanded or re-trained not only when changes in the environment or data are observed, but also when the model is required to include additional defect types. Thus, an important question is to explore the relationship between the size of the data collected and the performance of the system. This is particularly relevant for low occurrence defects, where collecting a large set of labeled data is not practical, at least before deployment. In this experiment, we start with the same base model and explore the effects of using 20%, 40%, 60%, 80%, and 100% increments of the labeled images from each defect-set for training. For example, for the 20% case, we randomly select 20% of the three datasets outlined in Section 4.5. After training, the model is tested on the same testing set used in Experiment 1.

**Figure 6:**
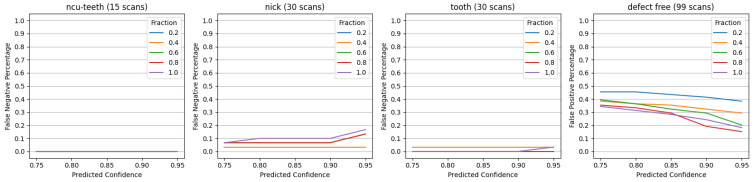
Prediction results using a consecutive image constraint of ϕ=3 and training with different fractions of data.

Figure 6 shows the results of training on different fractions of available data, using a consecutive image constraint of ϕ=3 from the results of Section 5.2. The NCU-Teeth defect can be seen to remain comparatively easy to identify, even when only 20% of the labeled data are used for training. On the other hand, for the Nick defect, increasing the amount of data had a negative effect on the false negative performance. It is possible that the model may have over-fit some of these samples. In the defect-free case, it can generally be seen that training on additional samples improves (decreases) the false positive detection rate.

### 5.4. Experiment 3: Generalization across Environments

**Figure 7:**
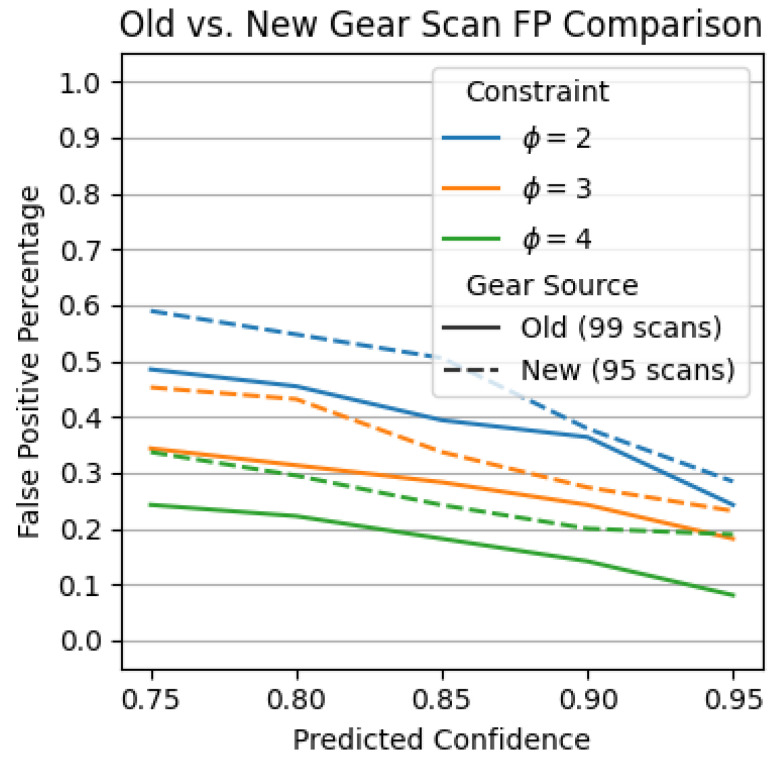
Direct comparison on model trained with old data on new defect-free gear scan sets. A lower score is better.

The data used in this work included different sets collected using different cameras with different intrinsic and extrinsic parameters, as mentioned in Section 4.5. While the new and old environments are similar, they are not exact. These changes also reflect real-world scenarios, where even if the exact same hardware and components are used, the imaging environment between any two inspection cells may not be exactly the same. In this experiment, we evaluate whether a multi-defect detection model trained on data collected in one environment (old) can generalize to data collected from another environment (new). Given the scarcity of defective parts, to enable a fair comparison, we evaluate these effects with respect to defect-free parts only and use the set of 99 and 95 gear scans described in Section 4.5. Figure 7 shows the inspection results.

From Experiment 1, the inspection parameters of ϕ=3 and p=0.9 were found to yield a balanced trade-off in FP and FN performance. Using these parameters, Figure 7 reports a slight increase in false positive detections, from 24.2% (old 99 scans, solid orange line) to 27.3% (new 95 scans, dashed orange line). Given the major revisions to the inspection system carried out in the work, these results are encouraging and suggest that only a small number of scans are likely to be missed during initial deployment prior to any additional fine-tuning.

## 6. Discussion

Defect inspection occurs as the images are collected by the system. The base rotation speed chosen in Section 3.3 represents the quickest possible speed; however, we found that when adding the detection algorithm to the system and measuring the performance on the 26-tooth gears (inspecting 52 images), the inspection process would require an additional 2.5 s to fully process all images in the scan. On average, this corresponds to approximately 0.144 s per image. The combined 7.5 s inspection time was found to meet the 10 s inspection time constraint imposed by the facility, without requiring further optimizations.

**Figure 8:**
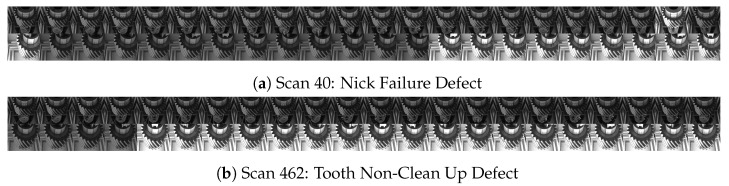
Sample scans with a defect, highlighting our inspection procedure. The top and bottom rows of (**a**,**b**) refer to the top and bottom cameras, respectively. The columns represent the gear tooth number. A bright image indicates a defect was predicted on that tooth with p≥0.75.

Figure 8 shows an example of the gear scanning process and how the inspection model interprets defect detection for a gear as a series of predictions for every tooth from every camera. The accuracy of the process is controlled by two parameters, *p* and ϕ. One aspect not explored in this work is a different constraint for each type of defect, which we expect may have more of an impact as additional defects are added to the system. We leave this for future work.

While we explored the impact of data size on performance, this was conducted only for the situation where all three defects were targeted for inspection at the same time. There are also other scenarios where, for example, the system could be trained to learn one defect at a time, which have not been explored in this work. It is possible that a facility may initially identify a frequent set of defects to be of high priority and later decide to add additional defects to the inspection. This scenario was left for future extension of this work.

## 7. Conclusions

In this work, we presented a real-time inspection system for industrial helical gears. The integrated system included both machine vision components and machine learning components. We explored how the design of the machine vision component can influence the performance of the machine learning component. We also considered various operational scenarios that can happen in production lines, such as using different datasets and data set sizes and the tradeoff between being very sensitive to detecting every defect and the rate of false positives. The presented system was developed and tested in a real manufacturing environment with gears having typical manufacturing defects. The results validate the design with several options available for further improvement.

## Figures and Tables

**Table 1:** Number of gear scans collected by our system. A gear may have multiple types of defects.

	Damaged Teeth	Nick	NCU	Other	No Defect
Gear Scans	408	510	179	255	766

**Table 2:** Number of training images and labeled instances.

	Damaged Teeth	Nick Defect	NCU Defect
Number of Images	1405 (2627)	2608 (3676)	324 (1896)

## Data Availability

Restrictions apply to the availability of data used in this paper. Data were obtained from an industrial partner and are available from the authors with the permission of the industrial partner.
